# Genome-Wide Identification Analysis of the *Rab11* Gene Family in *Gossypium hirsutum* and Its Expression Analysis in *Verticillium dahliae*

**DOI:** 10.3390/genes16080961

**Published:** 2025-08-14

**Authors:** Mengyuan Ma, Meng Zhao, Jiaxing Wang, Jianhang Zhang, Shuwei Qin, Ji Ke, Lvbing Fan, Wanting Yang, Wenjie Shen, Yaqian Lu, Mingqiang Bao, Aiping Cao, Hongbin Li, Asigul Ismayil

**Affiliations:** Ministry of Education Key Laboratory of Xinjiang Phytomedicine Resource Utilization, College of Life Sciences, Shihezi University, Shihezi 832000, China; mengyuanma2022@163.com (M.M.); zm022362@163.com (M.Z.); 18119249542@163.com (J.W.); zjh17630412656@163.com (J.Z.); qsw2623201516@163.com (S.Q.); 18062747918@163.com (J.K.); 17550355628@163.com (L.F.); 15138409277@163.com (W.Y.); 18919309525@163.com (W.S.); 18894489795@163.com (Y.L.); 20242006076@stu.shzu.edu.cn (M.B.); caoaiping@shzu.edu.cn (A.C.); lihb@shzu.edu.cn (H.L.)

**Keywords:** Rab11, *Gossypium hirsutum*, *Verticillium dahliae*, endocytosis

## Abstract

**Background/Objectives**: RAB11 (RABA) is a type of RAB GTPase. RAB GTPases are key components of membrane trafficking mechanisms, Rab11 is implicated in a variety of biological developmental processes and responses to biotic and abiotic stresses. Nevertheless, the role of Rab11 in the defense mechanisms of cotton against *Verticillium dahliae* (*V. dahliae*) remains to be elucidated. **Methods**: In the present study, by analyzing the transcriptome data of *Gossypium hirsutum* (*G. hirsutum*) infected with *V. dahliae*, in combination with gene ontology (GO) and Kyoto Encyclopedia of Genes and Genomes (KEGG) pathway analyses, the research focused on endocytosis. Further, through bioinformatics approaches, the endocytosis-related gene *Rab11* was identified. We conducted a genome-wide identification and analysis of *Rab11* in *G. hirsutum*. In addition, by integrating transcription factor (TF) prediction, prediction of protein–protein interactions (PPI) and quantitative real-time polymerase chain reaction (qRT-PCR), the gene expression of *Rab11* at different infection periods of *V. dahliae* (0, 24 and 72 hpi) were analyzed and validated. **Results**: The analysis of transcriptome data revealed that the endocytosis pathway is implicated in the stress response of cotton to *V. dahliae*. Additionally, three *Rab11* genes were identified as being involved in this stress response. Phylogenetic analysis revealed that the 65 genes in the *Rab11* family could be divided into four subgroups, each with similar gene structures and conserved motif patterns. **Conclusions**: The downregulation of *Rab11* in *G. hirsutum* is closely linked to its defense against *V. dahliae*. TF prediction coupled with PPI offers a roadmap for dissecting the signaling pathways, functional validation, and network construction of the three *GhRab11* genes.

## 1. Introduction

*Verticillium wilt* (VW), caused by the fungus *V. dahliae*, poses a significant threat to cotton production worldwide [1,2]. Owing to the intrinsic characteristics of *V. dahliae* [3], its host range is extremely wide, including over 200 economically crucial crops, especially cotton, eggplant, strawberry, tomato, and potato [4,5]. These crops are highly susceptible to VW, and, while significant advancements have been made in the identification of the pathogen, understanding its pathogenesis, and developing control strategies, the prevention and management of these diseases still face numerous challenges. Therefore, investigation of the molecular mechanisms underlying cotton VW not only facilitates the breeding of resistant cultivars but also provides valuable insights and potential solutions for enhancing the resistance of a wide range of economically important crops to *V. dahliae*.

In plant–pathogen interactions, vesicle trafficking plays a pivotal role in nearly all strategies of plant immune responses subsequent to pathogen attack. Intracellular vesicles mainly originate from endocytosis, whereas extracellular vehicles (EVs), which include exosomes and ectosomes, also constitute significant components of the cellular landscape [6]. Pathogens secrete vesicles laden with effector proteins, and, in response, plant cells also emit extracellular vehicles (EVs) into the apoplastic space. These EVs are rich in diverse bioactive molecules, including small RNAs (sRNAs), cell wall-degrading enzymes, and proteins that are responsive to both biotic and abiotic stresses [7]. During the intricate interplay between plants and fungi, the exchange of vesicles containing specific cargo is a common occurrence. Fungal pathogens exploit vesicle trafficking to deliver effector proteins into host cells, thereby manipulating the host’s immune responses to facilitate their colonization and infection [8]. When fungi form haustoria, plants transport vesicles to the haustorial site to deliver materials that form the haustorial membrane, thereby restricting pathogen invasion [9,10]. In the interaction between plants and pathogens, vesicle trafficking plays an essential role in plant immune responses, with a substantial portion of this trafficking being mediated by endocytosis. In this context, RAB GTPases are indispensable for vesicle transport, including the delivery of vesicular effector proteins to infection sites and their subsequent interactions among effector proteins [11,12]. Additionally, vesicle-associated membrane proteins in plants play a crucial role in resisting fungal pathogens [6].

Endocytosis is a highly conserved process in all eukaryotic organisms, facilitating the internalization of extracellular molecules into the cell through vesicles formed by the invagination of the plasma membrane [13]. This process is pivotal for plant cell signal transduction, intercellular communication, the establishment of cell polarity, and the maintenance of cellular homeostasis [14]. In plant cells, two primary forms of endocytosis have been characterized: clathrin-mediated endocytosis (CME) and clathrin-independent endocytosis (CIE). The latter category includes processes such as phagocytosis, macropinocytosis, and caveolin-dependent endocytosis [14,15,16].

In recent years, with the increasing research on vesicle trafficking mediated by Rab proteins, the gene families of various Rab GTPases in plants have been extensively studied. In eukaryotic cells, the plant endomembrane system is involved in various intracellular and intercellular biological functions, with vesicle trafficking serving as a crucial carrier for information exchange and material transport. RAB GTPases are key components of membrane trafficking mechanisms, functioning as molecular switches to regulate transport vesicles through reversible transitions between active and inactive states. It has been reported that the human genome encodes nearly 70 Rab GTPases, while the *Arabidopsis thaliana (A. thaliana)* genome encodes 57 Rab GTPases. In plants, the RAB members consist of eight categories: RABA, RABB, RABC, RABD, RABE, RABF, RABG, and RABH, corresponding to animal RAB 11, RAB 2, RAB 18, RAB 1, RAB 8, RAB 5, RAB 7, and RAB 6, respectively [17]. These eight groups are mostly conserved in green plants, with basal plants also harboring additional members, such as RAB23 [18,19]. Among them, RAB11 (RABA) is a conserved subfamily containing 26 RAB members, which are further divided into six subgroups (RABA1–RABA6), with RABA1 being the largest subgroup of RABA.

Rab11 (RABA) encompasses a diverse array of functions within plant cells, including involvement in apical growth, cytokinesis, pollen tube elongation, regulation of transport from the trans-Golgi network (TGN) to the plasma membrane, participation in endocytosis, maintenance of TGN morphology and function, and contribution to cell plate formation. In addition to these roles, Rab11 members modulate salt stress tolerance by participating in the trans-Golgi network and plasma membrane, are implicated in auxin signaling, and are engaged in polarized secretion in root hair cells [17,20]. These functions are crucial for the normal physiological activities of plant cells, particularly in response to environmental stimuli and during the regulation of cell division [21]. In addition to the aforementioned functions, research on Rab11 has revealed its close association with plant–pathogen interactions. The budded virions (BV) of Autographa californica multiple nucleopolyhedrovirus (AcMNPV) enter host cells via clathrin-mediated endocytosis. Studies have shown that RAB11 is involved in this process. Downregulation of Rab11 expression by dominant-negative (DN) mutation significantly reduced BV entry into Spodoptera frugiperda cells (Sf9), suggesting that plant Rab11-mediated endocytosis may be exploited by the virus BV as a means of host cell entry. However, the precise mechanism remains to be elucidated [22]. In contrast, in rice, transgenic plants overexpressing *OsRab11* exhibited resistance to bacterial pathogens such as Pseudomonas syringae pv. tomato DC3000, through the induction of jasmonic acid (JA)-responsive genes. This observation indicates that *OsRab11* is involved in the plant’s defense against pathogens [23].

## 2. Materials and Methods

### 2.1. RNA Sequencing Data Analysis

We obtained the raw RNA-seq reads and expression matrices (count values) for three different *G. hirsutum* cultivars—Zhongzhimian 2 (ZZM2), Junmian 1 (J1), and Xinluzao (XLZ)—that had been infected with *V. dahliae* from the VPI-MD database (https://www.bic.ac.cn/VPI-MD/#/Download, accessed on 22 May 2025) [24]. For each cultivar, three independent biological replicates (three different plants per time point) were sampled. After quality filtering, reads were aligned to the *G. hirsutum* reference genome with HISAT2. Abundance estimates in fragments per kilobase of transcript per million mapped reads (FPKM) were generated with StringTie. Differential expression analysis was performed in R (v4.3.2) using DESeq2. Transcripts exhibiting |log_2_FC| ≥ 1 and FDR < 0.05 (Benjamini–Hochberg) were designated as DEGs.

### 2.2. Weighted Gene Co-Expression Network Analysis and Fuzzy C-Means Clustering with Mfuzz

The weighted gene co-expression network analysis (WGCNA) was performed in WGCNA package (v1.73) [25]. The soft-thresholding power (β) was chosen as the lowest power that yielded a scale-free topology fit index (R^2^) ≥ 0.8. Modules were defined with a minimum size of 30 genes and module eigengene dissimilarity cut-off of 0.25. Prior to network construction, the expression matrix datExpr was log_2_-transformed (FPKM+1) and quality-filtered. A module–trait association was considered significant when the absolute correlation coefficient was ≥0.25 and the *p*-value was <0.05.

Fuzzy C-means clustering with Mfuzz (Mfuzz) was performed in Mfuzz package (v2.68.0) [26]. The genes (rows) with ≥25% missing values were removed, and remaining NAs were imputed with gene-wise means. Variables (columns) exhibiting a standard deviation of zero were removed to exclude non-informative features, and the data were z-score normalized (mean = 0, SD = 1). Soft clustering was performed using the fuzzy C-means algorithm, retaining data points with ACore ≥ 0.5 as significant genes.

### 2.3. Gene Ontology and KEGG Enrichment

Gene ontology (GO) (https://www.bic.ac.cn/VPI-MD/#/, accessed on 22 May 2025) and KEGG (https://www.bic.ac.cn/VPI-MD/#/, accessed on 22 May 2025) enrichment analyses were conducted to identify functional annotations related to metabolic pathways. Visualizations were generated using R package ggplot2 (v2.2.1). GO functional enrichment and KEGG pathway enrichment were considered significant at an adjusted *p*-value (adjP) < 0.05.

### 2.4. Genomic Identification of Rab11 Genes

*A. thaliana* genomic data and the *AtRAB11* reference sequence were downloaded from TAIR (https://www.arabidopsis.org, accessed on 22 May 2025). The whole-genome data of *G. barbadense* and *G. hirsutum* were retrieved from the NCBI website (https://www.ncbi.nlm.nih.gov/, accessed on 22 May 2025). After manually verified the conserved domain (PF00071) of the gene, we screened homologous genes in the whole-genome sequences of *G. hirsutum* and *G. barbadense* with the blast zone module in TBtools (Version 2.056). Resulting protein sequences were aligned in MEGA 11 software and a neighbor-joining(NJ) tree (1000 bootstrap replicates) was visualized and refined using the online platform iTOL: Interactive Tree Of Life (https://itol.embl.de/, accessed on 22 May 2025). Chromosomal positions were charted with TBtools (Version 2.056). Conserved domains and motifs were annotated with BioEdit (Version 7.0.9) and MEME (https://meme-suite.org/meme/, accessed on 24 May 2025) and visualized in TBtools (Version 2.056). Collinearity relationship was assessed via multiple collinearity scan (MCScanX) and dual synteny plotter. Putative cis-elements in Rab11 promoters were predicted with PlantCARE (https://bioinformatics.psb.ugent.be/webtools/plantcare/html/, accessed on 24 May 2025) and plotted in TBtools (Version 2.056). The physicochemical properties of 65 proteins in the *Rab11* family of *G. hirsutum* were analyzed using the “Protein Paramter Calc” function in TBtools (Version 2.056). The analysis yielded data on their relative molecular weights, isoelectric points, numbers of charged residues, instability indices, grand average hydropathicity, and aliphatic indices. For detailed information see Table S1 in Supplementary Materials.

### 2.5. TF Prediction and PPI Analysis of DEGs

TF Prediction is achieved by batch extracting protein sequences from the screened DEGs using the TbtoolsFasta Extract module based on gene IDs and then submitting them to the PlantTF database (PlantTFDB v5.0, https://planttfdb.gao-lab.org/prediction.php, accessed on 28 May 2025) for prediction. Protein–protein interaction (PPI) network analysis was conducted using the STRING database (https://cn.string-db.org/, accessed on 28 May 2025). The resultant networks were visualized using Cytoscape (v3.10.3) (https://cytoscape.org/, accessed on 28 May 2025). Additionally, the protein sequences of three differentially expressed genes (DEGs) were subjected to TF Prediction in the PlantTF database (Plant TFDB v5.0, https://planttfdb.gao-lab.org/prediction.php, accessed on 25 May 2025).

### 2.6. Plant and V. dahliae Materials

Roots of the disease-resistant cotton cultivar ZZM2, from the experimental fields of Shihezi University were collected at 0, 3, 6 and 24 hpi and stored at −80 °C before use. The *V. dahliae* strain V592, which was selected for this study, is deposited at the Ministry of Education Key Laboratory of Xinjiang Phytomedicine Resource Utilization, Xinjiang Production, College of Life Sciences, Shihezi University.

### 2.7. Real-Time Quantitative Polymerase Chain Reaction (qRT-PCR) Analysis

qRT-PCR primers were designed using the NCBI-primer blast tool (https://www.ncbi.nlm.nih.gov/tools/primer-blast/index.cgi?, accessed on 22 May 2025). Total RNA was extracted from three biological replicates using the RNA prep pure plant kit. Subsequently, 1 µL of RNA was reverse-transcribed into cDNA, with three technical replicates performed. The eIF-4α gene from *G. hirsutum* was used as the housekeeping genes. qRT-PCR was conducted using TianGen reagents on a Roche 480 system, with 40 cycles of amplification. Data were analyzed using the 2^−∆∆Ct^ method [27]. Primer sequences are listed in Table S2 of the Supplementary Materials, and gene sequences information is provided in Table S3 of the Supplementary Materials.

### 2.8. qRT-PCR Data Analysis

The experimental data were statistically analyzed using SPSS 22.0 software. The comparison of differences between groups was performed by one-way ANOVA. Origin 2024 software was used for graph drawing. The data were expressed as mean ± standard deviation (mean ± SD), and the experiment was repeated three times.

## 3. Results

### 3.1. Comprehensive Analysis of Downregulated Genes in G. hirsutum Following Infection with V. dahliae

We employed a comprehensive transcriptomic analysis approach, integrating Mfuzz, WGCNA, and RNA-seq analyses, to identify genes with similar expression patterns. By taking the intersection of these three analyses, we conducted a more thorough and in-depth exploration to identify key genes. This integrative approach provides valuable insights into the complex regulatory mechanisms of gene expression and their roles in biological processes.

In this study, transcriptome sequencing was performed on the resistant cultivar Zhongzhimian 2 (ZZM2) of *G. hirsutum* at three periods (0, 24, and 72 hpi) following infection with the *V. dahliae* (Vd592). Additionally, we conducted transcriptome sequencing and comparative analysis of ZZM2 at two periods (24 and 72 hpi) of infection with Vd592. Using the criteria of |Log2 (fold-change)| ≥ 1 and false discovery rate (FDR) < 0.05, a total of 8116 downregulated differentially expressed genes (DDEGs) were identified at the 24 and 72 hpi periods.

**Figure 1:**
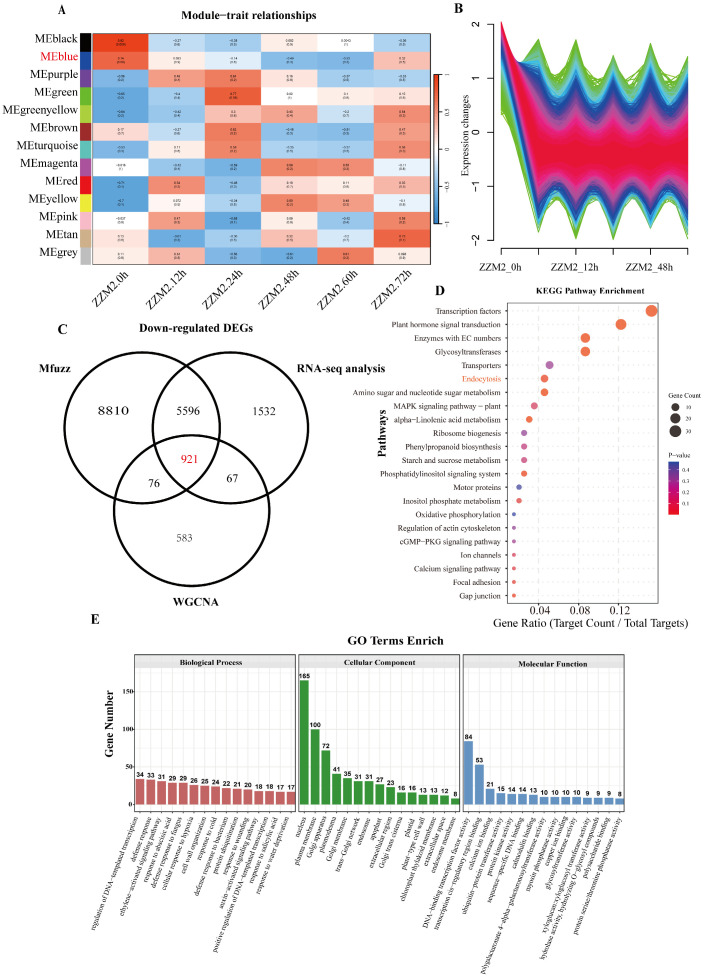
Comprehensive analysis of downregulated genes in ZZM2 infected by *V. dahliae*. (**A**) The transcriptome of ZZM2 infected by *V. dahliae*, (for each cultivar, three independent biological replicates were sampled) was analyzed using WGCNA to identify modules of co-expressed genes. The blue module was found to be closely related to the downregulated genes of interest; the *p*-value is shown in parentheses. (**B**) Gene expression patterns with periodic trends identified by Mfuzz. We obtained gene expression patterns with periodic upregulation/downregulation trends. The expression trends in this figure are closely related to the downregulated genes of interest; ACore ≥ 0.5. (**C**) Venn diagram showing overlaps among downregulated DEGs, Mfuzz, WGCNA, and RNA-seq analyses. The 921 genes in the intersection were identified as the focus of our study. (**D**) KEGG pathway analysis; (adjP) < 0.05. (**E**) GO Analysis; (adjP) < 0.05.

The transcriptome of ZZM2 at different infection periods after *V. dahliae* inoculation was analyzed by weighted gene co-expression network analysis (WGCNA) to identify co-expressed gene modules. WGCNA was employed to delineate the temporal associations between co-expression modules and the distinct infection periods of ZZM2, and to assign differentially expressed genes to their cognate modules. In this study, when the absolute correlation coefficient was ≥0.25 and the *p*-value was <0.05, the tissues were defined as tissue-specific modules, resulting in the identification of 13 tissue-specific modules. According to the expression of downregulated genes in the RNA-seq data, the blue module was found to be closely associated with the genes of interest. The final alignment yielded 1647 genes in the blue module (Figure 1A).

Fuzzy C-means clustering with Mfuzz (Mfuzz) was applied to analyze the expression trends of the transcriptome of ZZM2 infected by *V. dahliae* at different infection periods. After removing low-quality data, the fuzzy C-means algorithm was used to retain data points with ACore ≥ 0.5 as significant genes. The expression data were placed into a series of clusters. Based on the expression of downregulated genes in the RNA-seq data, we selected the clusters with the highest similarity in expression pattern (Figure 1B). As a result, a total of 15,403 genes were identified that exhibited high expression levels at 0 h, followed by a decrease in expression levels and stabilization.

Finally, the intersection of the DEGs obtained from RNA-seq, the 1647 genes from the WGCNA blue module, and the 15,403 genes from the Mfuzz analysis yielded 921 co-expressed genes.

The intersection of these three sets of genes yielded 921 DDEGs, which were subjected to KEGG analysis. The results indicate significant enrichment of transcription factors (TFs) and plant hormone signal transduction pathways, with a considerable number of genes involved (Figure 1C). Additionally, endocytosis-related genes also showed a certain degree of enrichment. GO analysis revealed that these genes were significantly enriched in biological process categories such as ethylene-activated signaling pathways, defense responses, responses to abscisic acid, defense responses to fungi, defense responses to bacteria, responses to cold, positive regulation of transcription from DNA template, responses to wounding, and auxin-activated signaling pathways (Figure 1D). These findings suggest that the molecular response mechanisms of cotton to VW may involve enhancing disease resistance through the regulation of ethylene signaling pathways, plant hormone signaling pathways, and gene transcription. KEGG analysis revealed that a large number of genes were involved in pathways including biological metabolism, signal transduction, transport proteins and endocytosis pathways (Figure 1E).

**Figure 2:**
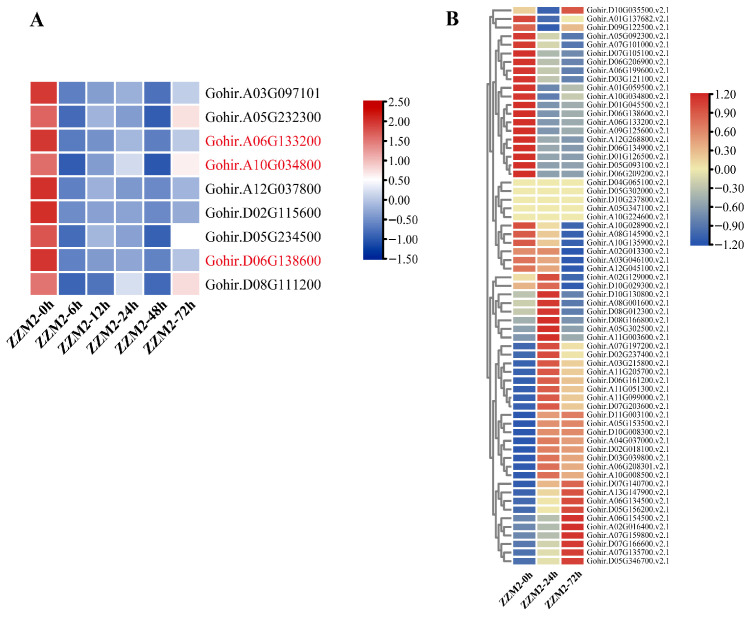
(**A**) Among the 921 genes, 9 were identified as being co-expressed with endocytosis-related genes, three of which belong to the *Rab11* family. (**B**) The heatmap illustrates the expression levels of the *Rab11* family genes in ZZM2 across three specific periods.

Further analysis focused on genes related to the endocytosis pathway. We identified nine genes that are both co-expressed and involved in the endocytosis pathway (Figure 2A). Upon gene identification, Gohir.A03G097101 and Gohir.D02G115600 were identified as uncharacterized proteins in *G. hirsutum*; Gohir.A05G232300 and Gohir.D05G234500 were identified as *G. hirsutum* phosphatidylinositol 4-phosphate 5-kinase 1 (GhPIP5K1); Gohir.A12G037800 was identified as *G. hirsutum* protein Early-responsive to dehydration 7 (GhERD7); and Gohir.D08G111200 was identified as *G. hirsutum* probable ADP-ribosylation factor GTPase-activating protein AGD6 (GhAGD6). Additionally, three of these genes were found to belong to the *G. hirsutum* ras-related protein RABA1c (*Rab11*) gene family and were renamed in this study as follows: Gohir.A06G133200 (GhRab11A06-1), Gohir.A10G034800 (GhRab11A10-3), and Gohir.D06G138600 (GhRab11D06-2). Subsequently, we conducted a comprehensive genome-wide identification of the *Rab11* family and further investigated the expression profiles of *Rab11* family genes in ZZM2 in response to infection by *V. dahliae*.

We analyzed the transcriptomic data of the *Rab11* family in ZZM2 at different periods of infection with *V. dahliae* and observed that the expression levels of some *Rab11* genes were downregulated (Figure 2B). To further elucidate the downregulation mechanism of *Rab11* genes, we conducted transcriptomic analyses of the *Rab11* family in three *G. hirsutum* cultivars, namely ZZM2, J1, and XLZ, at various periods post-infection with *V. dahliae* (see Figure S1 in Supplementary Materials). Interestingly, although all three cultivars belong to *G. hirsutum*, only the disease-resistant cultivar ZZM2 exhibited consistent downregulation of some *Rab11* family genes, by contrast, the susceptible cultivars J1 and XLZ showed no such pattern.

### 3.2. Identification and Features of Rab11 Genes in Cotton

**Figure 3:**
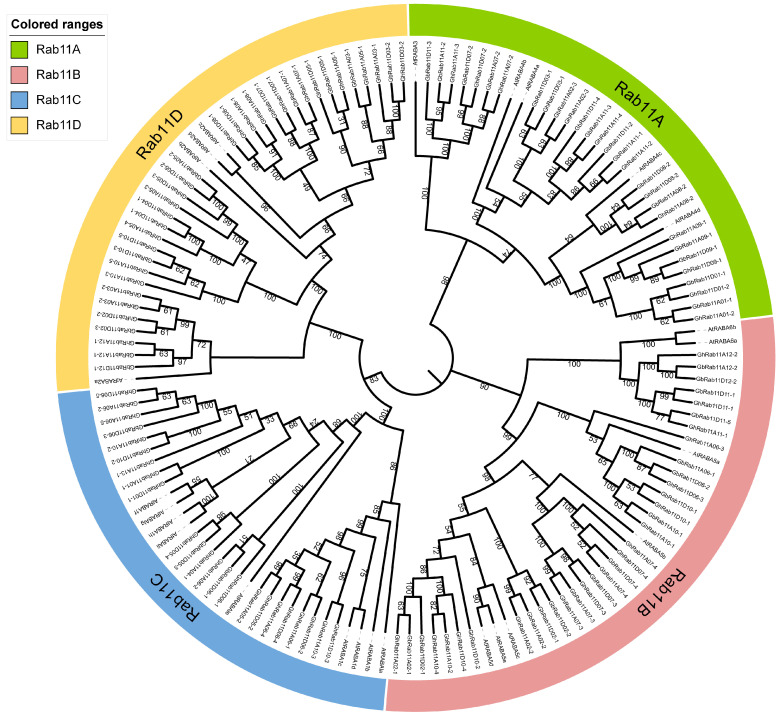
Phylogenetic relationships of *Rab11* in *G. hirsutum*, *A. thaliana*, *G. barbadense*. The neighbor-joining (NJ) phylogenetic tree depicts the evolutionary relationships of Rab11 proteins using MEGA X software with 1000 bootstrap replicates. The four subgroups examined are distinguished by various background colors.

To identify the *Rab11* genes in *G. hirsutum* and *G. barbadense*, we conducted a genome-wide prediction by performing BLAST analysis of the *Rab11* gene from *A. thaliana* using publicly available genome data. To investigate the evolutionary relationships of Rab11 proteins between *A. thaliana* and cotton, we used MEGA 11 software to compare the protein sequences and construct a neighbor-joining (NJ) tree with 1000 bootstrap replicates, as a result, a phylogenetic tree was obtained (Figure 3). The Poisson model was determined to be the most suitable for phylogenetic tree construction. The constructed phylogenetic tree revealed four distinct sub-clades, namely *Rab11A*, *Rab11B*, and *Rab11C*. We identified 65 *GhRab11* genes in *G. hirsutum* and 55 *GbRab11* genes in *G. barbadense*.

Given that *G. hirsutum* and *G. barbadense* are allo-tetraploids, their gene numbers far exceed those of *A. thaliana*, a simple diploid model plant. Driven by species-specific evolutionary differences and functional requirements, the Rab11 protein family, as a member of the small G-protein family, encompasses Rab11A, Rab11B, Rab11C, and Rab11D. These proteins play indispensable roles in intracellular membrane trafficking and are involved in various processes of cotton growth and development, such as cotton fiber development regulation, root tip polar growth, and stress resistance. However, it remains unclear which specific genes are involved in these mechanisms. In particular, there is a paucity of research on the Rab11 protein family in the resistant cotton cultivar ZZM2. We further analyzed the physicochemical properties of Rab11 proteins using the ExPASy website. The results (see Table S1 in Supplementary Materials) showed that the number of amino acids in *G. hirsutum* Rab11 proteins mostly ranged from 200 (GhRab11A13-1) to 236 (GhRab11D07-2), with molecular weights between 18.17 and 28.13 kDa. The predicted isoelectric points (pI) ranged from 4.83 (GhRab11D07-3) to 8.78 (GhRab11A02-3), indicating that the encoded Rab11 proteins may have higher solubility in neutral or weakly acidic environments. The instability index ranged from 17.05 (GhRab11A10-2) to 43.39 (GhRab11A10-5), with most values falling between 20 and 40. This suggests that these proteins may have a certain degree of stability in the cellular environment. Proteins with moderate instability indices may undergo dynamic structural changes within cells, making them suitable for biological processes that require rapid responses. The aliphatic index ranged from 75.53 (GhRab11D10-4) to 92.15 (GhRab11A10-5), with most values between 80 and 90. A higher aliphatic index indicates stronger hydrophobicity of the protein, which may confer greater stability in hydrophobic environments. The average hydrophobicity values ranged from −0.438 (GhRab11A02-1) to −0.173 (GhRab11D10-2), all of which were negative, indicating that these proteins are overall hydrophilic. These are likely to participate in water-soluble reactions within cells, such as enzyme catalysis and signal transduction.

### 3.3. Chromosomal Localization and Collinearity Relationship Analysis

**Figure 4:**
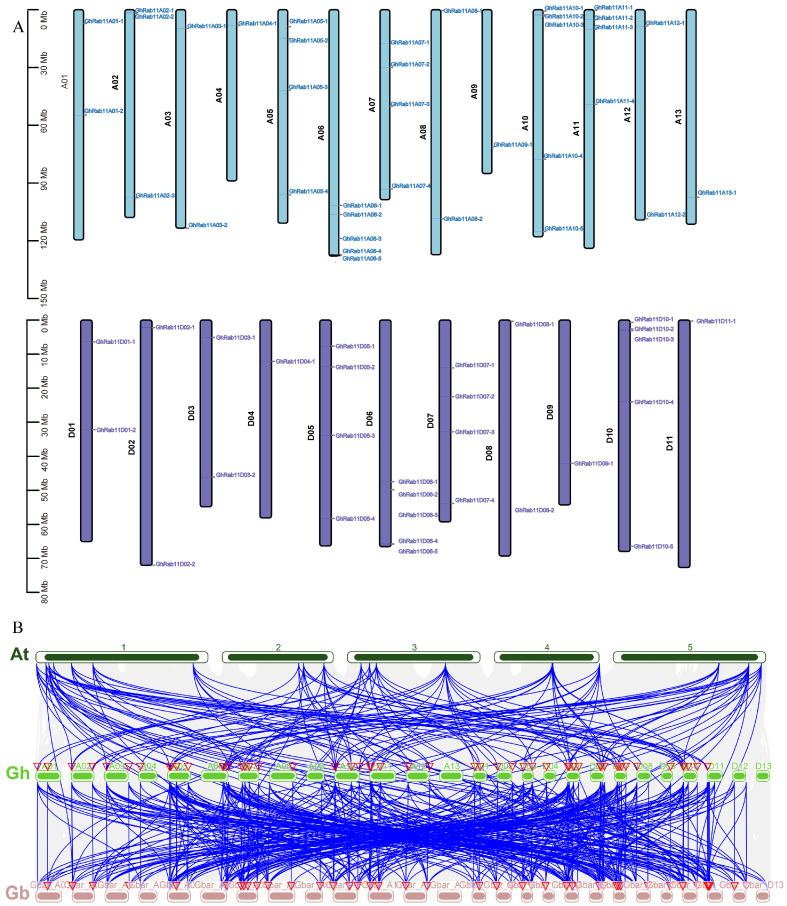
(**A**) Chromosome distribution of *Rab11* genes. (**B**) Collinearity relationship diagram of *Rab11* family genes among chromosomes of *A. thaliana*, *G. hirsutum*, and *G. barbadense*. At, Gh, and Gb represent *A. thaliana*, *G. hirsutum* and *G. barbadense*, respectively.

The chromosomal distribution of *GhRab11* is established based on genomic locations (Figure 4A). In *G. hirsutum*, a total of 65 *Rab11* genes are unevenly distributed across 24 chromosomes, with 13 on the A subgenome and 11 on the D subgenome. Specifically, 36 *GhRab11* genes are located on chromosomes of the A subgenome, and 29 *GhRab11* genes are situated on chromosomes of the D subgenome. The comparative genomic collinearity analysis of *A. thaliana*, *G. hirsutum*, and *G. barbadense* illustrates a high degree of collinearity between them *(*Figure 4B). Extensive collinear relationships were identified between chromosomes 1–5 of *A. thaliana* and the A and D subgenomes of both cotton species. Notably, the *Rab11* genes in *G. hirsutum* and *G. barbadense* exhibited a high degree of collinearity, further supporting the notion that they share a common ancestor and that their genomic structures have been largely conserved throughout evolution. Observations of multiple chromosomal translocations involving *Rab11* genes in both cotton species and *A. thaliana* suggest that these genes may have undergone frequent reorganization and selective pressures in response to long-term environmental adaptation. These translocation events could have facilitated the diversification of *Rab11* genes in cotton, enhancing their functional adaptability under varying environmental conditions. Additionally, these genomic alterations may be closely associated with the evolution of morphological, physiological, and reproductive traits in cotton, playing a pivotal role in the plant’s adaptation to changing environments. Further investigation into Rab11 will aid in a deeper understanding of the mechanisms by which *Rab11* genes have evolved in cotton and how they respond to and adapt to fluctuating environmental conditions.

### 3.4. Promoter Cis-Regulatory Elements and Gene Structure

**Figure 5:**
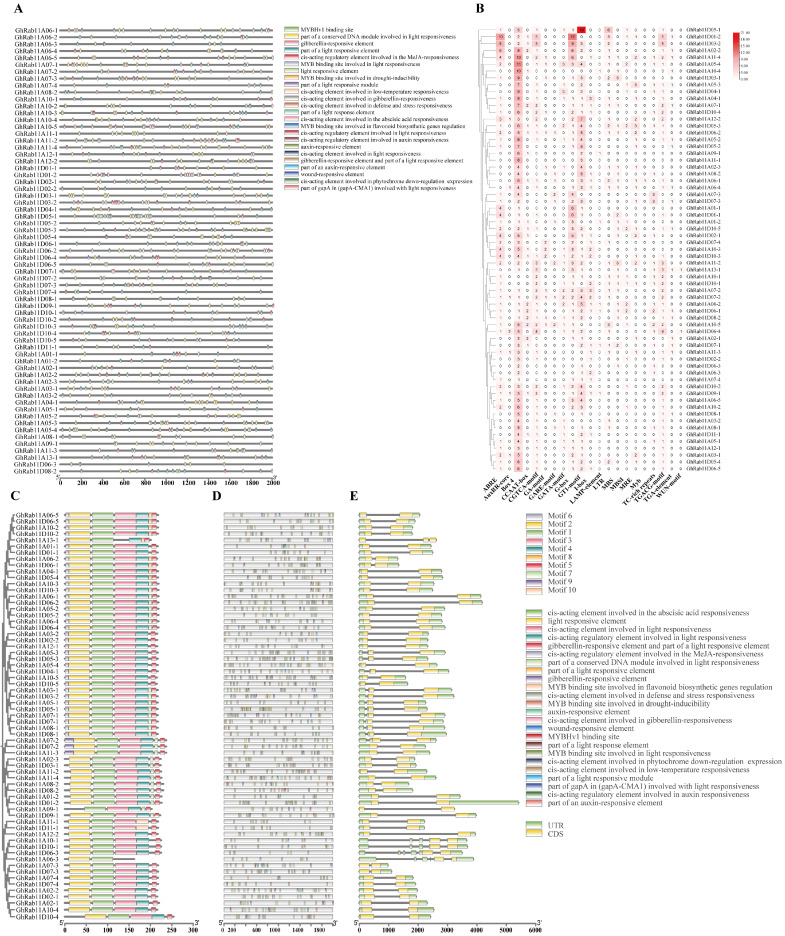
Analysis of promoter cis-acting element, conserved motif, and gene structure of *Rab11* family genes in *G. hirsutum*. (**A**) Analysis of the distribution of cis-acting elements in the *Rab11* promoter region. Different-colored ellipses represent different types of elements. The horizontal axis represents the base positions from the 5′ to the 3′ end of the promoter. (**B**) Number of cis-acting element category in the promoters of each *GhRab11*. The phylogenetic relationships among different *Rab11* genes were used to construct a phylogenetic tree. Based on this tree, the number of different cis-acting elements in the *Rab11* genes was represented in the form of a heatmap. The depth of the color was correlated with the quantity of the elements. The specific values are marked within the squares. (**C**) Based on this phylogenetic tree, a conservation analysis of the *Rab11* genes was performed. The differences in the colors of the squares represent the different types of motifs, while also intuitively presenting their positions within the genes. (**D**) Types and positions of cis-acting elements in *GhRab11*. Based on this phylogenetic tree, the positions of cis-acting elements within the *Rab11* genes are presented. (**E**) Based on this phylogenetic tree, the positions of introns and exons within the Rab11 genes are intuitively displayed. Green squares represent UTRs (including 5′ UTR and 3′ UTR), while yellow squares represent coding sequences (CDS).

We carried out a comprehensive investigation into the structural and functional variety of the *Rab11* gene family, examining aspects such as element distribution, evolutionary relationships, motif distribution, and CDS structure (Figure 5). By predicting the cis-acting elements and regulatory elements (CREs) in the *GhRab11* promoters and referring to Figure 5A,B,D, the following results were obtained. All *GhRab11* promoters contain a substantial number of light-responsive elements (including Box 4, G-box, GT1-motif, I-box, LAMP-element, TGACG-motif, WUN-motif, and GATA-motif). This finding strongly suggests that light signaling plays a pivotal role in regulating the expression of the *Rab11* gene family, which is likely closely associated with vital physiological processes such as photomorphogenesis and light-controlled growth in plants. In addition, we have identified numerous elements that are closely related to plant hormone responsiveness, including those responsive to gibberellin (GA), methyl jasmonate (MeJA), abscisic acid (ABA), and auxin (AuX). The majority of these promoters also harbor hormone-responsive elements, such as those responsive to gibberellin (GA-motif, GARE-motif), methyl jasmonate (TGA-element, CGTCA-motif), abscisic acid (ABRE), and auxin (AuxRR-core). As crucial signaling molecules within plants, plant hormones are indispensable in regulating various stages of plant growth and development as well as in responding to environmental stresses. The presence of these hormone-responsive elements indicates that the expression of the *Rab11* gene family may be finely regulated by multiple hormones, thereby participating in the complex physiological regulatory network of plants. In addition to the aforementioned elements, several other functional elements are present. The CCAAT-box, a promoter element, is one of the recognition and binding sites for RNA polymerase II, participating in the initiation and regulation of gene transcription. It is extensively found in the promoter regions of eukaryotic genes. The Myb site, as an MYB binding site, is involved in plant responses to a variety of signals, including light, hormones, and stress signals, and regulates the expression of genes related to plant growth and development as well as stress adaptation. The TC-rich repeats, acting as a silencer element, are involved in the negative regulation of gene expression, possibly by affecting chromatin structure to repress gene transcription. The long terminal repeat (LTR), mainly found in genomic elements such as retrotransposons, may play roles in genome structure and evolution and may also be involved in the regulation of gene expression. The presence of these elements provides strong molecular evidence for the potential functions of the *Rab11* gene family in enabling plants to cope with biotic and abiotic stresses. These elements may activate or repress the transcription of *Rab11* genes, allowing plants to rapidly adjust their physiological and metabolic processes under stress conditions, thereby enhancing their adaptability and stress resistance and ensuring their survival and reproduction in the complex and changing natural environment.

In the category related to abiotic and biotic stress responses, we have also identified a cultivar of elements that are closely connected to stress responses. For instance, elements involved in low-temperature responsiveness and those involved in defense and stress responses. The existence of these elements provides strong molecular evidence for the potential function of the *Rab11* gene family in enabling plants to cope with adverse environmental conditions such as drought, low temperature, and pathogen attacks. These elements may activate or repress the transcription of *Rab11* genes, allowing plants to rapidly adjust their physiological and metabolic processes under stress conditions, enhance their adaptability and stress resistance, and thus ensure their survival and reproduction in the complex and variable natural environment. Additionally, drought-induced MYB binding sites (MBS, MBSI) are present. The diversity of CREs in the *GhRab11* promoters suggests that these genes play important roles in regulating responses to various environmental conditions.

### 3.5. TF Prediction and PPI in G. hirsutum of DEGs

**Figure 6:**
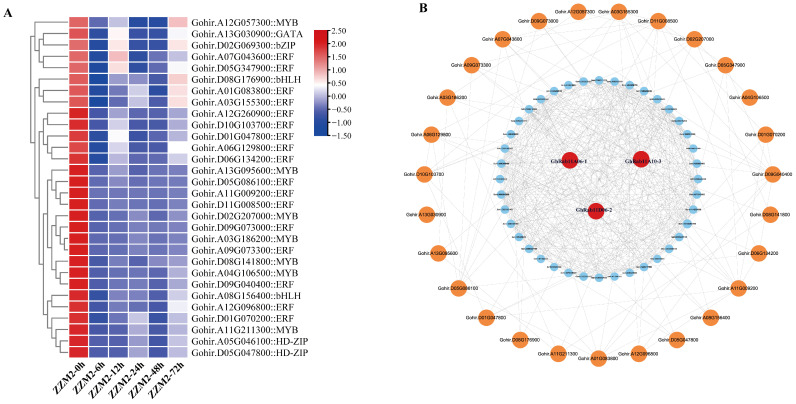
(**A**) The heatmap delineates the gene IDs and names of 30 transcription factors inferred from the protein sequences of three *Rab11* genes under investigation, as well as their gene expression levels across five periods (0, 6, 12, 24, 48, and 72 hpi) in ZZM2. (**B**) Protein–protein interaction (PPI) analysis of the three GhRab11. Red, orange, and blue colors represent GhRab11, the transcription factor of GhRab11, and other co-expressed genes, respectively. Hierarchical clustering analysis was performed using Euclidean distance and the complete linkage method.

In order to predict the functional modules of the three target genes, based on the mechanism that TFs function usually in clusters combined with PPI for functional module prediction studies, we performed TF prediction and PPI in the *G. hirsutum* of DEGs. Based on the comprehensive analysis of downregulated genes in *G. hirsutum* following infection with *V. dahliae*, The prediction results of the TFs are presented in the following heatmap (Figure 6A). Protein–protein interaction (PPI) prediction analysis was conducted using three genes—Gohir.A06G133200 (GhRab11A06-1), Gohir.A10G034800 (GhRab11A10-3), and Gohir.D06G138600 (GhRab11D06-2)—from the endocytosis pathways (Figure 6B). PPI predictive analysis has determined that a total of 30 genes do not directly interact with GhRab11, including GhPIP5K1 (phosphatidylinositol 4-phosphate 5-kinase 1) involved in phosphoinositide signaling, GhAGD6 (probable ADP-ribosylation factor GTPase-activating protein AGD6) associated with small GTPase regulation, GhERD7 (early-responsive to dehydration 7) and dehydration-responsive element-binding proteins—GhDREB1A and GhDREB1D-like—participating in dehydration stress responses, as well as four ethylene-responsive TFs—GhERF3, GhERF4, GhERF5, and GhERF024. Additionally, the interactome encompasses the G-box-binding factor GhGBF4 and members of the Myeloblastosis (MYB)—(GhMYB20, GhMYB30, GhMYB78, GhMYB106)—and Myelocytomatosis (MYC) (GhMYC2-like) transcription factor families. These interacting proteins provide critical targets for elucidating the molecular mechanisms underlying *G. hirsutum* defense against *V. dahliae*.

### 3.6. Validation of Expression Patterns of Rab11-Related Transcription Factors in G. hirsutum

**Figure 7:**
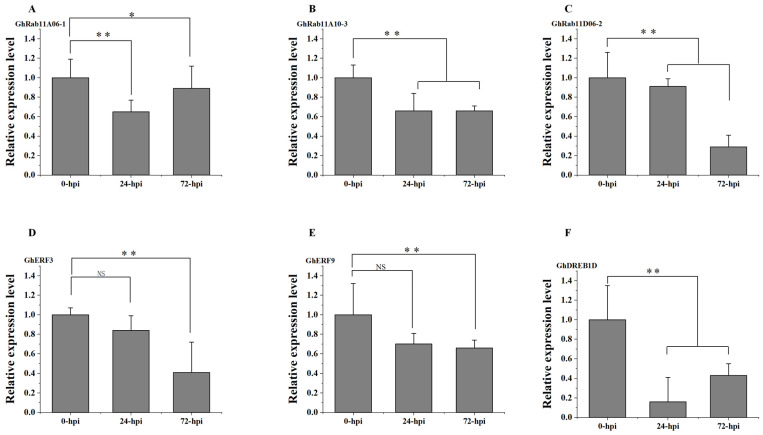
qRT-PCR validation of three candidate TFs and the three *Rab11* genes. The relative expression levels of three *Rab11* genes: GhRab11A06-1 (**A**), GhRab11A10-3 (**B**), GhRab11D06-2 (**C**) and three TFs GhERF3 (**D**), GhERF9 (**E**), GhDREB1D (**F**)—were determined by qRT-PCR in roots collected at 0, 24 and 72 hpi from ZZM2. The error bar represents standard deviation of three biological replicates. Data are mean ± SD, Student’s *t* test was applied to determine statistically significant differences (* *p* < 0.05; ** *p* < 0.001; *p* ≥ 0.05 not significant (NS)).

To verify the reliability of the transcriptomic data, we performed qRT-PCR validation on the expression levels of the three *Rab11* genes—GhRab11A06-1 (Figure 7A), GhRab11A10-3 (Figure 7B), and GhRab11D06-2 (Figure 7C)—and three TFs—Gohir.A01G083800 (GhERF3) (Figure 7D), Gohir.A03G155300 (GhERF9) (Figure 7E), and Gohir.A12G260900 (GhDREB1D) (Figure 7F)—which do not directly interact with the three *Rab11* genes. These results indicate that the relative expression levels of the three *Rab11s* and the three TFs at the three periods (0, 24 and 72 hpi) after *V. dahliae* infection are consistent with the transcriptome data analysis. Compared with 0 hpi, their expression levels were markedly reduced at both 24 hpi and 72 hpi.

## 4. Conclusions

Based on the transcriptome data analysis results of *G. hirsutum* in this study, *GhRab11,* in the resistant cotton cultivar ZZM2, exhibited a regular downregulation trend compared with susceptible cotton varieties. Through GO analysis and KEGG analysis of the downregulated genes, we found that the downregulated genes were closely related to endocytosis, we then identified nine endocytosis-associated genes among multiple downregulated genes with similar expression patterns, and verified that three of them consistently belonged to the *Rab11* family, which aroused our research interest in this discovery. Therefore, genome-wide identification analysis of *GhRab11* family and promoter cis-regulatory elements analysis showed that MBS and ABRE elements provided molecular evidence for the potential function of the *Rab11* gene family in plant response to adversities such as drought, low temperature, and pathogen attack. The results provide molecular evidence for the potential functions of the *Rab11* gene family in response to stresses such as drought, low temperature and pathogen attack in plants. The combination of TF prediction and PPI provides a reference for the further investigation of the major signaling pathways involved in the three target genes, as well as for subsequent functional validation and network construction.

## 5. Discussion

Combining the qRT-PCR validation experiments with the similar results of RNA-seq analyses, and given that genes with similar expression patterns may be functionally related or co-regulated in the pathway, we categorized the 30 genes predicted by the TFs to investigate the functions of the two types of TFs that accounted for a higher percentage of the TFs and found the following.

The ethylene-responsive TFs GhERF3, GhERF4, GhERF5, and GhERF024 belong to the ERF subfamily, with typical AP2/ERF structural domains [28]. These regulate target gene expression by recognizing GCC-box (AGCCGCC) or DRE/CRT elements. Most ERF TFs activate or repress the expression of downstream defense genes through positive or negative regulation, thereby enhancing plant responses to biotic and abiotic stresses. They play a key role in pathogen defense signaling. The potato StERF94 transcription factor belongs to the ERF family of proteins, and overexpression enhanced the expression of PR-associated defense genes, thereby enhancing potato resistance to *Fusarium solani* [29]. It has also been shown that potato StERF94 overexpression increased the tolerance of transgenic plants, especially in leaves, to drought, high temperature and combined stresses [30]. In cotton, GhERF1b is positively regulated by the GhMPK9-GhRAF39_1-GhWRKY40a module, which enhances the plant immune response by upregulating the expression of pathogenesis-related (PR) genes (PR1, PR2, PR3), and cotton is more susceptible to *V. dahliae* after silencing of GhERF1b by VIGS [31]. The ERF subfamily appears to increase plant disease resistance through positive regulation in *G. hirsutum–V. dahliae* interactions. However, the expression of GhERF subfamily genes fluctuates and decreases within 72 h of infection with *V. dahliae* in the disease-resistant cotton variety ZZM2, a phenomenon that deserves further in-depth study.

Promoter cis-regulatory element analysis combined with TF prediction revealed that the MYB binding site (MBS) is a drought-responsive element recognized by MYB TFs, and that MYB mediates the abscisic acid (ABA)-dependent drought response through the ABA-responsive element (ABRE). GhMYB20, GhMYB30, GhMYB78, GhMYB106 belong to the MYB TFs, which are a large gene family in plants and are widely involved in a variety of biological processes [32]. It has been shown by functional validation that GH_A11G1361 (GhMYB4) can participate in cotton phenol biosynthesis by regulating several key genes in the cotton phenol synthesis pathway [33]. Gossypol, a canonical terpenoid phytoalexin in *G. hirsutum*, is rapidly synthesized and accumulates upon pathogen challenge or environmental stress, exhibiting broad-spectrum antibacterial, antifungal, and antiviral activities. As a central component of the chemical defense arsenal against biotic stress in cotton, gossypol biosynthesis is therefore hypothesized to be modulated by MYB TFs to enhance resistance to *V. dahliae*. Nevertheless, this hypothesis remains to be rigorously tested.

Investigations into Rab11 have been extensively conducted in animals. Among Rab small G proteins, Rab11 directs the exocytic and recycling processes, thus controlling both the secretion and composition of plasma membrane [34]. In particular, Rab11 small GTPases are involved in the exocytic transport of lipids, receptors and transporters [35,36,37,38,39,40,41,42]. Given the crucial role of Rab11 in cellular transport processes, it has become a target for hijacking and exploitation by pathogens. Within the first line of defense, which is composed of epithelial and endothelial barriers, Bacillus anthracis and Cholera toxin disrupt the integrity of the membrane barrier and induce tissue disintegration by interfering with the Rab11-mediated transport of cell junction components [43,44]. During viral infections, the transport function of Rab11 is either exploited or subverted by pathogens. For instance, Salmonella typhimurium enhances its invasion efficiency by utilizing Rab11-mediated transport processes [45]. Hantavirus accomplishes viral budding or release by hijacking Rab11-mediated recycling endosomes [41,46]. In contrast, Shigella flexneri disrupts Rab11-mediated transport of antimicrobial peptides [47] and mediates an early transport defect interfering with the arrival of cargo at prevacuoles.

In the study of vacuolar transport pathways in tobacco leaf epidermal cells, it was found that mutant Rab11 mediates early transport defects, affecting the delivery of cargo to the prevacuolar compartment [48]. Concurrently, the Rab11 protein is essential for transport from the trans-Golgi network (TGN) to the plasma membrane (PM) [49]. The research on the interaction between Rab11 and pathogens in plants is still not fully adequate. However, based on some research conclusions, during the long-term coevolution of plants and pathogens, plants have continuously evolved complex defense mechanisms to resist pathogen invasion, while pathogens have correspondingly developed a cultivar of strategies to evade or disrupt the plant defense system. Among these, the Rab11 protein plays a key role in vesicular transport within plant cells. It is not only involved in the normal growth and development of plants and responses to abiotic stress, but also plays an important role in the interaction between plants and pathogens. Studies have shown that pathogens can interfere with the function of Rab11 by secreting effector proteins, thereby disrupting the plant immune response. For example, we found that Rab11 in plants interacts with the Phytophthora infestans effector RXLR24, during which the effector RXLR24 interferes with the secretion and transport of the host cell’s pathogenesis-related protein PR1, thereby disrupting the host immune response [21]. OsRab11 regulates the transport of intracellular vesicles to affect the transmission of JA-related signaling molecules, thereby activating the expression of downstream defense genes [23]. The interaction between pathogens and the Rab11 protein is an important aspect of the mutual game between plants and pathogens during their long-term coevolution, reflecting the complex molecular-level struggle between plants and pathogens.

Overall, especially in the study of biotic stresses on plants, *Rab11* has been attracting attention, and it is interesting to note that downregulation of *Rab11* expression in plant–virus interactions showed that plants were more resistant to disease, while plants overexpressing *OsRab11* in rice–fungus interactions were more resistant to disease. Therefore, we speculate that *Rab11* may play a “double-edged sword” role in plant–pathogen interactions. In this study, it was found that *Rab11* was affected by *V. dahliae*. This result, combined with the current research status of *Rab11* interaction between plants and pathogens, leads us to speculate that the function of plants in ZZM2—actively regulating the gene expression of *Rab11*—is reduced by *V. dahliae* infection. This reduction takes place in order to avoid this expression becoming a tool for pathogens to invade the host, instead becoming a source of disease resistance. The present study lays the foundation for elucidating the role of *Rab11* genes in VW resistance in cotton through the TF prediction and PPI of three *GhRab11* genes. In this study, the expression profile and potential regulatory network of the *GhRab11* family under VW stress were systematically mapped for the first time, which lays the foundation for elucidating its role in the vesicular transport–immunity cross-pathway, and provides candidate genes and regulatory elements for cotton disease resistance breeding, which is of great significance in providing a theoretical basis and genetic resources for cotton disease resistance breeding.

## 6. Limitations of the Study

This study has several limitations. First, no functional verification experiments were carried out, such as the downregulation or overexpression of *GhRab11* in ZZM2 by RNA interference (RNAi), CRISPR/Cas9 gene editing, or overexpression analysis to test its role in the process of resistance to yellow wilt in cotton. Secondly, the changes in protein level were not examined, and the experiment would have been more in-depth if the Rab11 protein expression had been detected by Western blot or ELISA to verify whether the changes in the transcript level were reflected in the protein level. Third, the subcellular localization of the Rab11 protein was not observed by fluorescent labeling (for example, Rab11: GFP fusion protein) in combination with confocal microscopy, and the next step of our study will be to elucidate the function of Rab11 protein in cytocytosis from this aspect. In the future, we will study the molecular mechanism of *GhRab11* in the interaction between *G. hirsutum* and *V. dahliae* from the above three aspects.

## Data Availability

All data generated or analyzed during this study are included in this article. Further inquiries can be directed to the corresponding authors.

## Supplementary Materials

The following supporting information can be downloaded at https://www.mdpi.com/article/10.3390/genes16080961/s1, Figure S1: Heatmap of *GhRab11* Gene Expression in J1 and XLZ Relative to ZZM2 under *V. dahliae* stress at 0, 24 and 72 hpi; Table S1: Basic information of Rab11 genes identified in *G. hirsutum*; Table S2: Primer sequences; Table S3: Gene sequences.
