# Supplementary material for: Genome-Wide Identification Analysis of the Rab11 Gene Family in Gossypium hirsutum and Its Expression Analysis in Verticillium dahliae
Source: Genes (Basel). 2025 Aug 14;16(8):961. doi: 10.3390/genes16080961 (PMC12385879; doi:10.3390/genes16080961)
Supplement: Supplementary file 1 [file genes-16-00961-s001.zip › genes-3777496-supplementary.pdf]

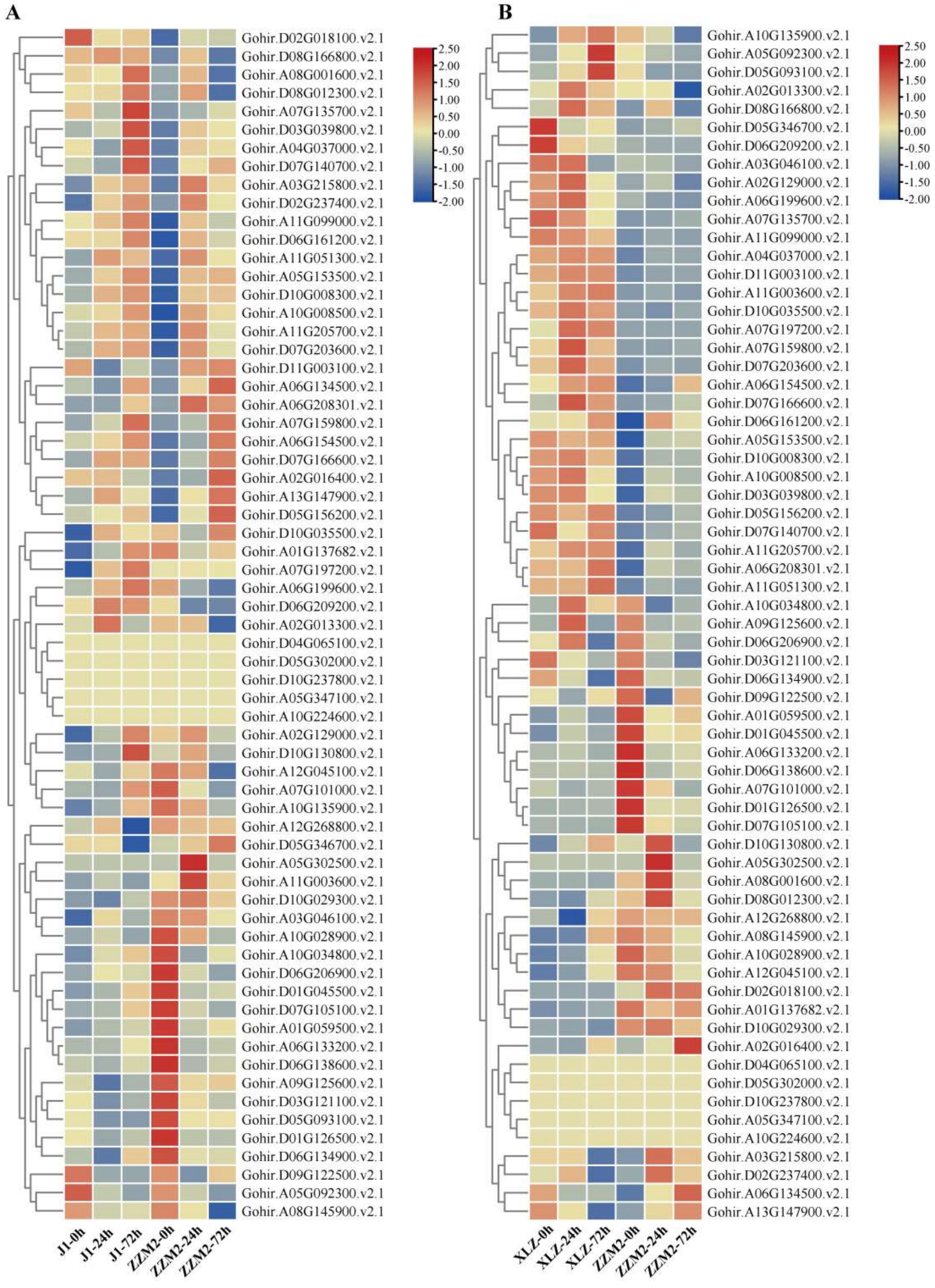

Figure S1. Heatmap of *GhRab11* Gene Expression in J1 and XLZ Relative to ZZM2 under *V.dahliae* stress at 0, 24 and 72 hpi. **(A)** The heatmap was constructed to illustrate the expression levels of the *GhRab11* family genes at three periods (0, 24, and 72 hpi) in response to *V. dahliae* infection in the cotton cultivars J1 and ZZM2. **(B)** The heatmap was generated to depict the expression levels of the *GhRab11* family genes at three periods (0, 24, and 72 hpi) in the cotton cultivars XLZ and ZZM2 during infection with *V. dahliae*.

TableS1.Basic information of *Rab11* genes identified in *G. hirsutum*

Sequence ID	Number of Amino Acid	Molecular Weight	Theoretical pI	Instability Index	Aliphatic Index	Grand Average of Hydropathi-city
Gohir.A01G059500.1.v2.1	216	24195.41	5.87	26.02	91.2	- 0.259
Gohir.A01G137682.1.v2.1	225	24950.27	5.95	35.65	86.27	- 0.265
Gohir.A02G013300.1.v2.1	219	24526.48	5.37	32.99	77.44	- 0.438
Gohir.A02G016400.1.v2.1	215	24122.9	5.03	36.43	84.79	- 0.318
Gohir.A02G129000.1.v2.1	223	24544.95	8.78	33.11	86.64	- 0.267
Gohir.A03G046100.1.v2.1	216	23935.02	6.22	41.16	86.76	- 0.26
Gohir.A03G215800.1.v2.1	215	23819	6.76	42.41	85.35	- 0.321
Gohir.A04G037000.1.v2.1	217	24209.2	5.85	23.52	82.67	- 0.299
Gohir.A05G092300.1.v2.1	216	23946	6.23	37.18	84.54	- 0.298
Gohir.A05G153500.1.v2.1	218	24093.15	5.49	25.92	84.54	- 0.25
Gohir.A05G302500.1.v2.1	216	24037.21	6.32	42.09	86.3	- 0.263
Gohir.A05G347100.1.v2.1	216	24085.29	6.44	33.1	90.37	- 0.237
Gohir.A06G133200.1.v2.1	218	24130.14	5.5	26.98	81.83	- 0.309
Gohir.A06G134500.1.v2.1	215	23951.12	5.48	33.36	89.35	- 0.242
Gohir.A06G154500.1.v2.1	161	18171.62	6.14	31.8	90.87	- 0.3
Gohir.A06G199600.1.v2.1	218	24187.3	5.49	27.5	81.42	- 0.307
Gohir.A06G208301.1.v2.1	217	24010.1	5.98	22.32	87.65	- 0.231
Gohir.A07G101000.1.v2.1	216	23826	7.01	33.27	84.95	- 0.263
Gohir.A07G135700.1.v2.1	236	25971.38	5	26.56	81.4	- 0.249
Gohir.A07G159800.1.v2.1	217	24249.15	4.84	38.04	82.21	- 0.351
Gohir.A07G197200.1.v2.1	217	24022.86	5	34.04	81.34	- 0.334
Gohir.A08G001600.2.v2.1	216	23769.68	5.89	38.87	84.95	- 0.284
Gohir.A08G145900.1.v2.1	229	25157.53	6.84	42.15	86.86	- 0.187
Gohir.A09G125600.2.v2.1	202	22475.66	5.55	33.06	90.3	- 0.183
Gohir.A10G008500.1.v2.1	224	24689.76	5.1	42.57	80.98	- 0.296
Gohir.A10G028900.1.v2.1	217	23817.88	5.97	17.05	88.06	- 0.183
Gohir.A10G034800.1.v2.1	218	24007.03	5.35	22.76	83.62	- 0.251
Gohir.A10G135900.1.v2.1	215	24029.88	5.25	25.89	79.81	- 0.411
Gohir.A10G224600.1.v2.1	214	23669.83	6.32	43.39	92.15	- 0.184
Gohir.A11G003600.1.v2.1	217	24666.18	6.02	38.14	84.01	- 0.322
Gohir.A11G051300.1.v2.1	224	24674.02	6.33	35.08	88.84	- 0.213
Gohir.A11G099000.2.v2.1	236	26166.77	5.24	23.77	88.43	- 0.19
Gohir.A11G205700.1.v2.1	225	24876.27	6.34	35.25	86.27	- 0.286
Gohir.A12G045100.1.v2.1	215	23876.05	6.76	42.29	83.07	- 0.371
Gohir.A12G268800.1.v2.1	216	24465.68	5.53	38.32	86.2	- 0.342
Gohir.A13G147900.2.v2.1	200	22151	5.61	20.71	88.2	- 0.181

Gohir.D01G045500.1.v2.1	216	24223.46	6.13	26.37	91.2	- 0.263
Gohir.D01G126500.1.v2.1	225	24964.34	6.62	33.32	86.27	- 0.267
Gohir.D02G018100.1.v2.1	215	24157.94	4.96	37.42	84.33	- 0.346
Gohir.D02G237400.1.v2.1	215	23835.04	6.76	41.52	87.16	- 0.296
Gohir.D03G039800.1.v2.1	223	24574.97	8.78	35.76	86.64	- 0.266
Gohir.D03G121100.1.v2.1	216	23860.88	6.22	39.26	86.76	- 0.259
Gohir.D04G065100.1.v2.1	216	24070.34	6.91	31.11	90.37	- 0.214
Gohir.D05G093100.1.v2.1	216	23974.06	6.53	37.18	84.54	- 0.303
Gohir.D05G156200.1.v2.1	218	24111.18	5.49	26.31	82.75	- 0.261
Gohir.D05G302000.1.v2.1	216	24037.21	6.32	42.09	86.3	- 0.263
Gohir.D05G346700.1.v2.1	217	24255.34	6.53	28.39	87.14	- 0.275
Gohir.D06G134900.1.v2.1	215	23832.09	6.45	34.23	88	- 0.239
Gohir.D06G138600.1.v2.1	218	24268.33	5.5	30.73	80.05	- 0.317
Gohir.D06G161200.2.v2.1	224	24704.82	5.24	42.26	81.43	- 0.321
Gohir.D06G206900.1.v2.1	218	24141.21	5.49	25.1	82.29	- 0.298
Gohir.D06G209200.1.v2.1	217	23980.08	5.98	22.32	88.11	- 0.219
Gohir.D07G105100.1.v2.1	216	23854.05	7.01	34.16	85.42	- 0.262
Gohir.D07G140700.1.v2.1	236	25881.28	5.09	26.21	81.02	- 0.244
Gohir.D07G166600.1.v2.1	217	24226.06	4.83	38.89	81.75	- 0.361
Gohir.D07G203600.1.v2.1	217	24022.86	4.99	34.24	81.8	- 0.336
Gohir.D08G012300.1.v2.1	216	23840.76	5.89	38.91	84.95	- 0.31
Gohir.D08G166800.2.v2.1	229	25156.55	7.73	42.15	86.86	- 0.187
Gohir.D09G122500.1.v2.1	222	24898.21	5.6	28.42	82.16	- 0.3
Gohir.D10G008300.1.v2.1	224	24690.75	5.01	42.91	80.98	- 0.296
Gohir.D10G029300.1.v2.1	217	23902	5.7	18.33	89.86	- 0.173
Gohir.D10G035500.1.v2.1	218	24037.06	5.36	22.91	83.17	- 0.263
Gohir.D10G130800.1.v2.1	253	28132.58	6.01	30.18	75.53	- 0.37
Gohir.D10G237800.1.v2.1	214	23669.83	6.32	43.39	92.15	- 0.184
Gohir.D11G003100.1.v2.1	217	24785.3	5.77	39.9	84.01	- 0.324

TableS2. Primer sequences

**Sequence ID**	**Gene name**	**Primer name**	**Primer sequences (5'–3')**	**Primer usage**
Gohir.A06G133200.1.v2.1	GhRab11 A06-1	GhRab11-1-F	AAGATGGGAAATCCTTCGCGG	qRT-PCR
		GhRab11-1-R	CATAGCTGACACGTCTTTACCCAC	
Gohir.A10G034800.1.v2.1	GhRab11 A10-3	GhRab11-2-F	GGGACACTGCTGGTCAAGAA	qRT-PCR
		GhRab11-2-R	TGAGACAGCCACAAGGTGAC	
Gohir.D06G138600.1.v2.1	GhRab11 D06-2	GhRab11-3-F	GTGGCTGTCTCAACCGAAGA	qRT-PCR
		GhRab11-1-R	AGCTGACACGTCTTTACTGACA	
Gohir.A01G083800.1.v2.1	GhERF3	GhERF3-F	GCAACCCGGCGGATCTATTA	qRT-PCR
		GhERF3-R	TTCGAAAGGAAAAGCCGGGA	
Gohir.A03G155300.1.v2.1	GhERF9	GhERF9-F	GAAAGAAAAGCCGTGTTTGGC	qRT-PCR
		GhERF9-R	CCTACTGCCCCGGTCAC	
Gohir.A12G260900.1.v2.1	GhDREB1D	GhDREB1D-F	AATCCCGGGAAGTGGGTTTC	qRT-PCR
		GhDREB1D-R	TTCTGAAAGTCTCCGCCACC	
GheIF-4α		GheIF-4α-F	ACATGGACCAGAACACTCGT	qRT-PCR
		GheIF-4α-R	AACCTTCCACTTCGTCCGAT	

TableS3. Gene sequences

**Sequence ID**	**Gene name**	**Gene sequences (5'–3')**
Gohir.A06G133200.1.v2.1	GhRab11 A06-1	ATGGCTGGTTACAGAGCTGAGGATGACTATGACTATCTTTTCAAGGTGGTTTT-<br>GATCGGTGATTCCGGTGTCGGAAAGTCAAATCTGCTCTCGAGGTTCACCAGGAAC<br>GAGTTTAGCCTCGAGTCCAAGTCCACTATTGGTGTTGAGTTCGCTACAC-<br>GTAGCTT-<br>GAATGTTGATGGAAAGGTCATCAAGGCTCAGATTTGGGACACTGCTGGTCAAGA<br>AAGGTATCGTGCCATAACAAGTGCCTATTATCGAGGAGCTGTTGGTGCACTTCTT-<br>GTG-<br>TATGATGTTACACGACACTCCACATTCGAAAATGTCGAGAGGTGGTTAAGAGAG<br>TTGAGGGATCACACAGATCCCAACATTGTAGTCATGCTCATTGGTAA-<br>TAAATCCGATCTTCGTCACCTTGTGGCTGTCTCAACCGAAGATGGGAAATCCTTC<br>GCGGAGAAAGAATCCCTCTACTTCATGGAAACTTCTGCCCTGGAAGCTACT-<br>AATGTT-<br>GAAAATGCATTTGCCGAAGTTCTGACACAGATCTACCATATCGTGAGCAAGAAA<br>GCTATGGAGACCAGCGAGGAAGGGAATGCTTCAGCTGTCCCATCAAAGGGAGA-<br>GAAAATCGATGTGGGTAAAGACGTGTCAGCTATGAAGAAGGGAGGTTGCTGCTC<br>AAGCTAG
Gohir.A10G034800.1.v2.1	GhRab11 A10-3	ATGGCTGGTTACAGAGCAGAGGATGACTATGACTACCTTTTCAAGGTAGTTTT-<br>GATCGGTGATTCAGGTGTGGGGAAGTCTAATTTACTCTCCAGGTTCACCAGGAAC<br>GAGTTCAGCCTCGAGTCCAAGTCTACTATCGGCGTTGAGTTCGCTACTCGTAG-<br>TTT-<br>GAATGTGGATGGCAAGGTCATTAAAGCTCAGATTTGGGACACTGCTGGTCAAGA<br>AAGGTACCGTGCAATAACAAGTGCCTATTACCGAGGAGCTGTGGGAGCAC-<br>TCCTTGTGTAC-<br>GACGTTACACGCCACTCCACATTCGAAAACGTAGAGAGGTGGCTAAGAGAGTTG<br>AGGGATCACACAGATCCCAACATCGTAGTCATGCTTGTCG-<br>GAAACAAATCAGATCTTCGTCACCTTGTGGCTGTCTCAACCGACGATGGGAAATC<br>CTTTGCTGAGAAAGAATCCCTTTACTTCATGGAAACTTCTGCTCTGGAAGCTACT-<br>AACGTG-<br>GAAATTGCATTTGCTGAAGTTCTTACTCAGATATACAACATCGTTAGCAAGAAAG<br>CTATGGAGACAAGCGATGATGGGGCCGCTTCAGCCGTGCCCTCCAAGGGAGA-<br>GAAA<br>ATTGATGTCGGTAAAGATGTCTCGGCAATGAAGAAAGGGGGTTGCTGTTCAA-<br>GCTGA
Gohir.D06G138600.1.v2.1	GhRab11 D06-2	ATGTCTGGTTACAGAGCTGAGGATGACTATGACTATCTTTTCAAGGTGGTTTT-<br>GATCGGTGATTCCGGAGTCGGAAAGTCAAATCTGCTCTCGAGGTTCACCAGGAA<br>CGAGTTTAGCCTCGAGTCCAAGTCCACTATTGGTGTTGAGTTCGCTACTCG-<br>TAGCTT-<br>GAATGTTGATGGAAAGGTCATCAAGGCTCAGATTTGGGACACTGCTGGTCAAGA<br>AAGGTATCGTGCCATAACAAGTGCCTATTATCGAGGAGCTGTTGGTGCACTTCTT-

		GTG-<br>TATGATGTTACACGACACTCCACATTCGAAAATGTAGAGAGGTGGTTAAGAGAG<br>TTGAGGGATCACACAGATCCCAACATTGTAGTCATGCTCATTGGTAA-<br>TAAATCCGATCTTCGTCACCTTGTGGCTGTCTCAACCGAAGATGGGAAATCCTTC<br>GCGGAGAAAGAATTCCTCTACTTCATGGAAACTTCTGCCCTGGAAGCTACT-<br>AATGTCGAAAATGCATTTGCCGAAGTTCTGACACAGATCTACCATATCATGAGCA<br>AGAAAGCTATGGAGACCAGCGAG-<br>GAAGGGAATGCTTCAGCTGTCCCATCGAAGGGAGAGAAA<br>ATCGATGTCAGTAAAGACGTGTCAGCTATGAAGAAGGGAGGTTGCTGCTCAA-<br>GCTAG
Gohir.A01G083800.1.v2.1	GhERF3	ATGAGGAGAGGGAGAGGTGCCGCAGCGGCGGCTCCTGCCGCCGCGGCAAAC-<br>GCCGTAGCTAG-<br>GAGACCGGCACTGCAACCCGGCGGATCTATTAAAGAGCCGAGATACAGAGGTGT<br>TAGAAAAAGGCCATGGGGCAGATTCGCGGCCGAGATTCGAGACCCCTGGAA-<br>GAAGAC-<br>CAGGGTCTGGTTAGGGACGTTCGACTCAGCCGAAGAAGCCGCTCGAGCCTACGA<br>TACGGCGGCGAGGACACTCCGTGGACCCAAAGCTAAAACAAATTTCCCCA-<br>TAAATTCTTCAAATATCCCGGCTTTTCCTTTCGAAACCAATCATCACCACAACGA<br>AGGGTTCATCGACCAACGCCGGTTATATCCGATGGGCGAT-<br>TTTCATGACCCCGAAGTGAATCCACAGAGACCCACGAGGAGTAGCATGAGTAGC<br>ACGGTGGAATCGTTTAGTGGACCCAGACCGGTCCAACCACCG-<br>CAAAAATCGGCG-<br>GACTTCGCGGTGGTTTCGACTAGGAAGTACTATCCGAGGCCGCCGCCAGTAGAG<br>CCAGAGGATTGTCATAGTGACTGTGATTCATCATCGTCGGTGGTTGATGATGGG-<br>GA-<br>TATCGCGTTGTCTTCGTGTCGGAAAACTTTGCCTTTCGATCTCAATTTTCCACCCTT<br>GGATGAAGATGAAGATCTCCAGTGTACCGCTTTATGTCTTTGA
Gohir.A03G155300.1.v2.1	GhERF9	ATGGCTCCCCAAGACAAAAATGCGAGCAAAATCTTGAAGAAAGCTAAC-<br>GTTACTGGAAGTAC-<br>GAGCAGCCAAGAGGTGCATTTCAGGGGAGTAAGGAAGAGGCCATGGGGTAGGT<br>ACGCTGCCGAAATCAGAGATCCCGGAAAGAAAAGCCGTGTTTGGCTTGG-<br>TACTTTCGATAC-<br>GGCTGAGGAAGCTGCCAGAGCCTACGACGCGGCGGCGCGTGAGTTTCGTGGACC<br>TAAGGCTAAGACCAACTTCCCTTTACCGGATGAAAC-<br>CAACTGTTACAAGGGCCAGAACCAG-<br>CAGAGCCCTAGCCAAAGCAGCACGGTAGAGGAATCTGGAAGCCCAACGGTGGA<br>GCGTGGAGTCAACACTGTGACCGGGGCAGTAGGGAGAT-<br>TCCCTTTCGCGTGCCACCAG-<br>CAGCTGGCTCTGGGTGGTGGAGTCGCTAATGGTGGGATTAGCGGGGTGACCCAG<br>TCGCGGCCGGTTCTATTTATCGAAGCGTTGGGAGGAGCTGGCGTTGTT-<br>GGTCAGGTTTATCCGGTTCGGTTCGATCCGGTGGGAGTGCAGTTGGGTATGGGAT<br>TTGCAAGTGTTGTCCGAAGTGAACCGGACTCTTCATCGGCCATTCATT-<br>GCAAGGCAAGGA-<br>GACCTGGCCTTGTCCTCGATCTTAACCTTCCTCCTCCAGTCGATGCTTGA

Gohir.A12G260900.1.v2.1	GhDREB1D	ATGGTTGATTCTGGGTCGGTTTCTGAAAGTG-GAACTGATCGTCCGGTGAATTTTTCCGATGAATATGTGATGTTAGCTTCGAGTTATCCAAAGAGGCGAGCTGGGAGGAAGAAGTTCCGGGAGACTCGACACCCGGTG-TACCGTGGAG-TTCGCCGGAGGAATCCCGGGAAGTGGGTTTCTGAAGTGAGGGAGCCTAATAAGAAGTCGAGGATTTGGCTTGGAACTTTCCCGACGGCGGA-TATGGCGGCGCGTGCTCACGAC-GTGGCAGCTATAGCACTGAGAGGGAAGTCAGCTTGTTTGAACTTCGCTGACTCAGCTTGGAATCTTCCGGTCCCGGCTTCTTCCGACCCAAAGGATATTCAAAAGAC-GGCGGCG-GAGGTGGCGGAGACTTTCAGAACGGCTGAGTGTTCGTGCGGGAATTCTAGAAACGATTCAAAGAGAAGTGAAAACACGGAGATGGAGAAAGGGTTTTATTTGGAC-GAAGAAGCGTT-GTTTGGGACACAAAGATTTTGGGCAAATATGGCTGCCGGTATGATGATGTCACCTCCTCGTTCCGGTCATGACGGAGGATGGGAAGAACATGAAGTAGATGAT-TATGTACCTTTATGGAGTTATTCTATTTAA
